# Association of *SYNE1* locus with bipolar disorder in Chinese population

**DOI:** 10.1186/s41065-019-0095-7

**Published:** 2019-06-17

**Authors:** Wenqiang Li, Yongfeng Yang, Binbin Luo, Yan Zhang, Xueqin Song, Ming Li, Luxian Lv

**Affiliations:** 1 Department of Psychiatry, Henan Mental Hospital, The Second Affiliated Hospital of Xinxiang Medical University, Xinxiang, Henan China; 2 Henan Key Lab of Biological Psychiatry, Xinxiang Medical University, Xinxiang, Henan China; 3 The First Affiliated Hospital of Zhengzhou University, Zhengzhou, Henan China; 4 Key Laboratory of Animal Models and Human Disease Mechanisms of the Chinese Academy of Sciences and Yunnan Province, Kunming Institute of Zoology, Chinese Academy of Sciences, Kunming, Yunnan China; 5 Henan Province People’s Hospital, Zhengzhou, Henan China

**Keywords:** *SYNE1*, rs9371601, Genome-wide association study, Bipolar disorder, DNA methylation

## Abstract

**Objectives:**

Genome-wide association studies (GWAS) suggest that rs9371601 in the *SYNE1* gene is a risk SNP for bipolar disorder (BPD) in populations of European ancestry, but further replication analyses across distinct populations are needed.

**Methods:**

We analyzed the association between rs9371601 and BPD in a Han Chinese sample of 1315 BPD cases and 1956 controls.

**Results:**

We observed a significant association between rs9371601 and BPD in Han Chinese (*p* = 0.0121, OR = 0.859). However, further examinations revealed that the Europeans and Chinese subjects had different BPD risk alleles at the locus. We then found that rs9371601 had different “minor alleles” and distinct linkage disequilibrium (LD) patterns surrounding itself in Europeans and Han Chinese, which might be the explanation of the observed inconsistent association signals for this locus in different populations. Our explorative analyses of the biological impact of rs9371601 suggested that this SNP was significantly associated with the methylation of a CpG site (cg01844274, *p* = 5.05⨯10^− 6^) within *SYNE1* in human dorsal lateral prefrontal cortex (DLPFC) tissues.

**Conclusions:**

Our data confirms the association between rs9371601 and BPD, but the underlying biological mechanism remains to be fully elucidated in further studies.

**Electronic supplementary material:**

The online version of this article (10.1186/s41065-019-0095-7) contains supplementary material, which is available to authorized users.

## Introduction

Bipolar disorder (BPD) is a severe neuropsychiatric disorder with a lifetime prevalence of ~ 0.75% worldwide [1, 2]. Recent studies have revealed relatively high heritability of BPD [3]. Also, genetic analyses including genome-wide association studies (GWAS) have reported many common genetic variations showing moderate to strong associations with BPD, and these variants have been repeatedly highlighted in subsequent analyses of enlarged sample sizes [4–15]. One epic study in the field of BPD genetics was the meta-analysis of multiple BPD GWAS datasets followed by independent replications by the Bipolar Disorder Working Group of Psychiatric Genomics Consortium (PGC1) in 2011 [13]. In this study, loci at *CACNA1C*, *ODZ4* and several other genomic areas exhibited genome-wide significant associations with BPD (*p* < 5.0⨯10^− 8^). Following this, the PGC2 scientists recently performed a larger GWAS with multiple European cohorts (29,764 cases and 169,118 non-psychiatric controls), and identified 30 genomic loci showing genome-wide significant associations with BPD, which span genes including *TRANK1*, *ANK3*, *NCAN* and *ITIH3* [15]. These discoveries quickly caught wide attention and have elicited numerous replicative studies.

Majority of the follow-up efforts have been put into understanding whether genes highlighted in these GWAS are indeed susceptibility genes of BPD in populations other than Europeans [16–21], and such cross-population replications were not limited to BPD [22–25]. For example, Gonzalez et al. conducted replication analysis of the European GWAS risk loci in a Latino BPD cohort [16], and Zeng et al. replicated the associations between *DGKH* SNPs and haplotypes with BPD in Chinese populations [18]. While many of the highlighted genes (e.g., *ANK3*) have been replicated [20], whether *SYNE1*, a promising BPD susceptibility gene identified in GWAS by PGC1 [13, 26] and also found to confer the risk of BPD in other populations, remains to be examined. The discovery of *SYNE1* as a potential risk gene for BPD was led by the identification of genome-wide significant association between a single nucleotide polymorphism (SNP) rs9371601 within this gene and the illness in the discovery stage of the aforementioned GWAS by Green et al. (7481 BPD individuals and 9250 control individuals) [13]. Although this association was not replicated in their replication samples, they have successfully validated the significant association between rs9371601 and BPD susceptibility in an independent sample from the Great Britain (1527 cases and 1579 controls) [26]. Further meta-analysis combining their data with the previous GWAS samples confirmed the genome-wide level significance of this association signal [26]. In the PGC2 BPD GWAS study including 29,764 cases and 169,118 controls, while rs9371601 did not achieve genome-wide level of statistical significance, it showed nominal associations in both the discovery and replication samples with the same direction of allelic effects (discovery, *p* = 1.80⨯10^− 6^; replication, *p* = 0.0175) [15]. In addition to the genetic analysis of rs9371601, functional studies of its associated BPD candidate risk gene, *SYNE1*, have emerged. The human *SYNE1* gene contains 145 exons and encodes multiple proteins. A recent study has investigated one of its proteins, CPG2 [27]. CPG2 is brain-specific and primarily locates in the excitatory postsynaptic areas to exert impact on synaptic plasticity and function [27–29]. Rathje et al. found that the protein expression of CPG2 was significantly decreased in the postmortem brains of BPD cases versus control subjects, and was regulated by genetic variants in its promoter and coding regions [27]. Taken together, dissecting the roles of BPD risk variation and *SYNE1* in the pathogenesis of the disease may provide valuable insights.

Since rs9371601 was discovered as a BPD risk SNP in European populations, we have explored the recent two GWAS in East Asians [8, 30] to examine its link with BPD in other populations in the world. While this SNP was not highlighted in these two cohorts, further investigations are needed before conclusions can be made regarding its role in BPD in Eastern Asians. We have recruited a BPD case-control sample from Mainland China and sought to test whether rs9371601 was also associated with BPD in these subjects. Additionally, we have analyzed the impact of rs9371601 on gene expression and DNA methylation using public datasets. Comparisons of allelic frequencies and LD patterns of the genomic regions encompassing 9,371,601 between Europeans and Chinese were also performed.

## Material and methods

### BPD case-control samples

1315 BPD patients of Han Chinese origin were recruited from several provinces of Mainland China (e.g., Henan, Hunan, Shanghai, Zhejiang and Sichuan). Part of the samples have been previously described elsewhere [21, 31]. To minimize the impact of confounding variables, patients were excluded if they (i) had a history of mental retardation, drug/alcohol abuse, or schizophrenia; or (ii) had comorbid diagnosis of other brain injury. Diagnoses were confirmed by a research psychiatrist via an Extensive Clinical Interview and a Structured Clinical Interview for DSM-IV Axis/Disorders, Patient Version (SCID-P). For control subjects, 1956 individuals were recruited from local communities, and those with any history of major mental illnesses, neurological disorders, or a family history of severe forms of brain disorders were excluded from further participation. The study protocol was approved by the ethics committee of the Second Affiliated Hospital of Xinxiang Medical University and the ethics committees of all participating hospitals and institutes. All participants provided written informed consents before any study-related procedures were performed.

### SNP genotyping

Genomic DNA samples of the participants were isolated from their peripheral blood leukocytes using high salt extraction method. The SNP was genotyped using the SNaPShot method. Briefly, the genomic fragments containing rs9371601 were amplified from 20 ng genomic DNA in a 15 μL volume of polymerase chain reaction (PCR) reaction in 96-well plates. The amplified PCR fragments were purified by treatment of SAP and Exo-I, and specifically designed SNaPShot primers were used to amplify the SNP target sites. After one base extension, the reaction was terminated and the products were loaded on an ABI 3730 automatic sequencer to generate SNP genotype callings, which were automatically performed using GeneMarker V2.2.0 and manually verified. All genotypes were called blind to sample identity and affection status.

### Statistical analysis

Hardy-Weinberg equilibrium (HWE) test was conducted using the “Haploview” software [32]. The program PLINK (v1.07) was used to calculate *p*-values, odds ratios (OR) and 95% confidence intervals (CI) using the “logistic regression” option [33], with sex and region of participants included in the covariates. We also conducted a power analysis of the current sample size using the “Power and Sample Size” program [34] to examine whether it had sufficient statistical power. Linkage disequilibrium (LD) between rs9371601 and its nearby SNPs was analyzed in 1000 Genomes Project Phase 1 genotype data using tools on the SNAP website.

### Queries about the impact of rs9371601

To investigate the potential impact of rs9371601, we examined the association between this SNP and the mRNA expression of nearby genes in brain and blood tissues using several expression quantitative trait loci (eQTL) databases. In brief, we first examined rs9371601 in Brain xQTL dataset (http://mostafavilab.stat.ubc.ca/xQTLServe/) [35] containing the RNA-seq results of human dorsal lateral prefrontal cortex (DLPFC) tissues and relevant genome-wide SNP genotyping of the donors. According to the original report, the researchers performed polyA+ RNA-seq in the DLPFC of 494 European individuals. Detailed information, including the sample information, statistical methods, and the strategies taken to minimize impact of known and hidden confounding factors, can be found in the original publication [35]. We also utilized the GTEx dataset of genome-wide SNP information and whole transcriptome RNA-seq data in frontal cortex (BA9) (https://www.gtexportal.org/home/) [36] for the current analyses. The GTEx contains RNA-seq data from multiple postmortem brain regions of primarily non-psychiatric Europeans, providing valuable eQTL information. Herein we utilized the eQTL data in frontal cortex tissues collected from 118 donors (BA9). The details for sample collection, data processing and statistical analyses can be found in the original study [36]. Since many eQTL are usually population specific, and the above brain eQTL datasets mainly contain non-Asian samples, we have also utilized a blood eQTL dataset consisting of East Asian donors to understand the role of rs9371601 in this population. We extracted eQTL data of 85 East Asian individuals (including Han Chinese and Japanese) and 56 European subjects from Stranger study [37], in which genome-wide mRNA expression in lymphoblastoid cell lines from HapMap3 global populations was analyzed using the microarray technology. Details of the demographic characteristics of participants, mRNA quantification methods and statistical analyses can be found in the original report [37]. In addition, we also investigated the impact of rs9371601 on DNA methylation using the Brain xQTL dataset [35], in which global DNA methylation (420,103 methylation sites) was quantified using 450 K Illumina array in 468 European individuals. The Spearman’s rank correlation was used to calculate the association between rs9371601 and DNA methylation status. Detailed information regarding this methylation quantitative trait loci (meQTL) can be found in the original report [35].

## Results

In our case-control sample, we obtained ideal genotyping call rate for rs9371601. Further analyses showed that this SNP was in Hardy-Weinberg Equilibrium (*P* > 0.05) in both cases and controls. According to the allelic frequencies of rs9371601 in Han Chinese (data from 1000 Genomes Project [38]) and the reported ORs of this SNP in European GWAS [15, 26], we performed power analyses of the current sample, and found that the current sample size had sufficient statistical power (> 80%) for the purpose of this study. In the previous European GWAS analyses [13, 15, 26], the T-allele of rs9371601 was significantly overrepresented in BPD patients compared with controls (0.364 in cases and 0.349 in controls, Table 1), making it a “risk” allele for BPD. However, the results obtained from our Han Chinese case-control sample were in contrast to that in Europeans, as the frequency of T-allele was significantly lower in 1315 BPD patients than that in 1956 non-psychiatric controls (0.743 in cases and 0.773 in controls, *p* = 0.0121, OR = 0.859, Table 1).

**Table 1:** Association of rs9371601[T-allele] with bipolar disorder in world populations

Sample	Ethnics	Case	Control	Fre_Case	Fre_Control	*P*-value	OR	95%CIs
Chinese	Chinese	1315	1956	0.743	0.773	0.0121	0.859	0.763–0.967
PGC2 discovery [15]	European	20,352	31,358	0.364	0.349	1.80⨯10^−6^	1.069	1.040–1.098
PGC2 replication [15]	European	9412	137,760	/	/	0.0175	1.049	1.009–1.092

*Fre* frequency, *OR* odds ratio, *CIs* confidence intervals

**Fig. 1:**
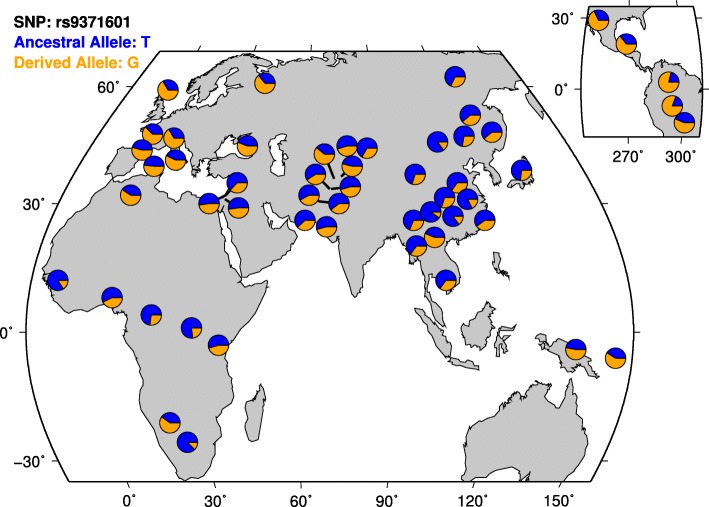
Global distributions of rs9371601 in 53 world populations from HGDP dataset [39, 40]

The opposite directions of allelic effects for rs9371601 in European and Han Chinese populations, which might seem odd, were intriguing. To explore possible explanation of this inconsistency, we examined the characteristics of genetic architectures for this SNP in both populations, and found that it was always the “minor allele” conferring genetic risk of BPD in both Europeans and Chinese, however, the “minor allele” at rs9371601 was T-allele in the Europeans, while in Han Chinese, its “minor allele” switched to G-allele. This observation was made using data of the 1000 Genomes Project [38], in which we saw that the frequency of rs9371601 T-allele was 0.371 in European populations but 0.716 in Han Chinese subjects. This result was reproduced using another database from HGDP (http://hgdp.uchicago.edu/) worldwide populations [39, 40] (Fig. 1).

**Fig. 2:**
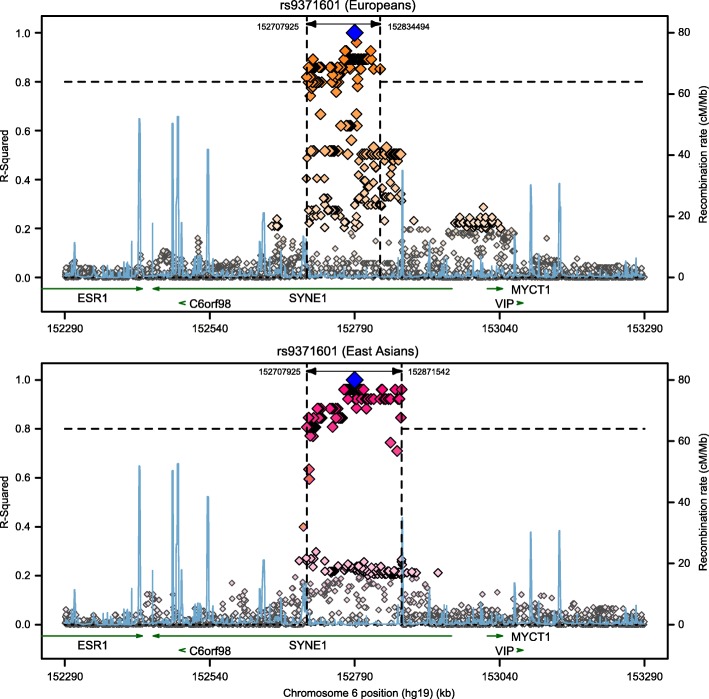
Comparisons of rs9371601 LD SNPs between European and East Asian populations

We also performed LD analysis for rs9371601 using the SNAP website, and identified numerous SNPs in high LD (*r*^*2*^ > 0.8) with this SNP in both European and East Asian populations (Fig. 2). However, there were observable differences between its LD patterns in these two populations. For example, there were 128 SNPs in high LD with rs9371601 in East Asians (*r*^*2*^ > 0.8), whereas in Europeans, 34 of these 128 SNPs were not highlighted in the pool of show strong LD SNPs for rs9371601 (*r*^*2*^ < 0.8) (Additional file 1: Table S1).

Although the BPD risk alleles at rs9371601 in distinct populations are different, previous studies and our findings strongly suggest involvement of this SNP in the genetic risk of BPD. Investigating the potential biological impact conferred by different alleles at rs9371601 is therefore necessary and valuable to understand mechanisms underlying the pathological risk of BPD linked to this locus. It is the consensus that risk SNPs of complex psychiatric disorders usually affect gene expression or epigenetic modifications in brain and blood tissues [41–45], we thus tested whether rs9371601 was an eQTL or meQTL of particular gene(s) or DNA methylation sites. In the brain eQTL datasets that comprised of European individuals (i.e., Brain xQTL [35] and GTEx [36]), rs9371601 was not associated with the mRNA expression of any gene in its surrounding regions (Additional file 2: Tables S2 and Additional file 3: Table S3). In the blood eQTL databases containing European or East Asian subjects [37], rs9371601 was neither associated with the expression of any nearby genes (Additional file 4: Tables S4 and Additional file 5: Table S5). It should be noted that our analyses have been mainly focused on the mRNA expression levels of annotated genes, and the possibility that this SNP is associated with the mRNA levels of specific exons or isoforms in certain genes, or even some previously uncharacterized RNAs, was not tested. Considering that *SYNE1*, the gene most likely affected by rs9371601, is a large gene containing many exons and undergoes extensive alternative splicing processes (Additional file 6: Figure S1), further studies analyzing the association between rs9371601 and mRNA levels of different transcripts of *SYNE1* are necessary, given that accumulating studies have proven the important roles of alternative splicing in the pathogenesis of psychiatric disorders [44, 46, 47]. In addition, we also carried out meQTL analyses for rs9371601, and found that this SNP was significantly associated with the methylation of a CpG site (cg01844274, *p* = 5.05⨯10^− 6^) within *SYNE1* in the DLPFC region in Brain xQTL dataset (Additional file 7: Table S6) [35]. While the functional consequences of the methylation at this site is still unclear, this result is in line with the recent studies showing that psychiatric risk SNPs are usually associated with DNA methylation in human brains [42, 48, 49].

## Discussion

Genetic analysis of BPD has been emerging in recent years, and GWAS is a popular approach that has implicated many risk loci for this illness. Along with these discoveries, genes such as *TRANK1*, *ANK3*, *NCAN* and *ODZ4* have been presumed to be conferring genetic risk of BPD in European populations [15]. However, it is widely accepted that genetic heterogeneity exists among different populations [50, 51], and genetic markers of a illness identified in one population do not always confer such risk in other populations. Therefore, the genetic risk architecture of BPD in Han Chinese, which has been relatively understudied comparing with that in Europeans, remains to be tackled. Despite the previous achievements in genetic analyses of BPD in Han Chinese [30], replication of reported risk loci in Europeans has been a major task. However, owning to the limited sample sizes in early GWAS of East Asians [30], few loci with robust statistical associations with BPD in these populations have been found. We therefore recruited BPD case and control subjects so as to provide insights into the genetic architecture of BPD in Han Chinese.

Our current study has examined whether the SNP rs9371601 is a potential BPD risk marker in Han Chinese, and implicated important roles of *SYNE1* in this illness. The *SYNE1* gene was initially reported in a genome-wide meta-analysis of diverse European GWAS datasets, and one of its intronic SNP rs9371601 was genome-wide significantly associated with BPD in the discovery sample. Although this SNP was not significantly associated with BPD in the replication stage of the above GWAS [13], it was found nominally associated with BPD in British populations in a later follow-up study, and meta-analysis combining this sample and all available previous data yielded a genome-wide significant association signal of this locus with BPD [26]. In the latest PGC2 BPD GWAS, the significant association between rs9371601 and BPD was again implicated. However, in this GWAS, rs9371601 did not achieve genome-wide level of statistical significance, though very close to the threshold [15]. These lines of evidence suggest that rs9371601 is likely a risk locus for BPD at least in European populations. In the present study using a relatively large sample of Han Chinese populations, while we did observe a significant association between this SNP and the illness, the allelic effect was in the opposite direction compared with that in Europeans. This is not the first case that a specific locus shows varied association trends with an illness in distinct populations. For example, rs6265 in *BDNF* was reported to confer risk of BPD in Europeans but not in Asians [52]. However, it is intriguing that the “minor” allele of rs9371601 in Europeans is different from that in Han Chinese populations, providing possible explanations for the inconsistent allelic effects discussed earlier. Given that risk genes for one psychiatric disorder usually exhibit robust associations with multiple other psychiatric illnesses as well [53–55], researchers have also conducted a cross-diagnosis analysis rs9371601. This SNP was nominally associated with major depressive disorder (MDD) in the Green study of European individuals, and the T-allele (i.e., BPD risk allele in Europeans) predicted increased risk of MDD [26]. We have also examined this SNP in the GWAS of schizophrenia from European populations, and the T-allele was again associated with an increased risk (*p* = 0.0033, OR = 1.030) [56]. Intriguingly, in consistent with our BPD analysis in Han Chinese, the frequency of T-allele at rs9371601 was again lower in Chinese MDD patients versus controls from a recent GWAS, although the association did not achieve nominal significance (*p* = 0.493, OR = 0.971) [57]. Therefore, rs9731601 is likely an authentic risk marker for an array of psychiatric illnesses, with distinct genetic risk architectures between Europeans and Chinese.

We have also explored the possible biological impact of the BPD risk allele at rs9371601 in the brain. By performing LD analysis, we found that there were multiple SNPs in high LD with rs9371601 in at least one population. However, eQTL analysis did not identify any association signals between this SNP and the expression of nearby genes, adding more difficulties in the elucidation of the underlying molecular mechanisms for the involvement of rs9371601 in BPD. We also examined the mRNA expression of *SYNE1* from recent transcriptome datasets of DLPFC tissues from BPD patients and controls, and the total mRNA expression of *SYNE1* did not differ significantly between cases and controls. Several *SYNE1* isoforms showed reduced expression in BPD patients versus controls, but such association signals did not survive multiple correction [58]. This result is also partially consistent with that of a previous study [27]. Nevertheless, our meQTL analyses using the Brain xQTL dataset [35] showed that rs9371601 was significantly associated with the methylation of a CpG site (cg01844274, *p* = 5.05⨯10^− 6^) in *SYNE1*, suggesting that this SNP (or its linked variants) may exert function at the epigenomic level. Given the differences in population history and genetic architectures between Europeans and East Asians, there is also the possibility that the overall risk associations or directions in this specific genomic region are different between these two populations, but the possibility is not denied that there could be the unidentified causative variant(s) with the same risk direction in both populations. In the potential event that there is the undiscovered causative variant(s), the inconsistency of linkage between rs9371601 and BPD could be resulted in the disrupted LD connection between rs9371601 and the unidentified causative variant(s) owning to the differences in the LD structures in this genomic region between Europeans and East Asians.

Our study has presented the first replication analysis of *SYNE1* rs9371601 in Han Chinese population. Despite the observed significant association between this SNP and the illness, and the insights into its biological impact, certain limitations should be acknowledged. First, this study does not confirm the reasons of the inconsistent association signals at this SNP with BPD between Europeans and Asians. We speculate that the differences of LD structures in this genomic region might be the explanation, and further fine-scale mapping of these genetic markers are necessary to prove this hypothesis. Second, the current analyses did not take into consideration the potential genetic background differences between various BPD subtypes (BP-I, BP-II or BP-NOS) in Han Chinese individuals, as rs9371601 showed associations with all types of BPD in Europeans [15]. Future studies stratify different BPD subtypes might be of great interest. Third, although we identified a meQTL association of rs9371601 in the brain DLPFC, the underlying regulative mechanisms of such effect is unknown. To the best of our knowledge, the functional connections underpinning meQTL associations have been a hassle in the relevant field, and the functional consequences of the DNA methylation are yet to be identified.

## Additional files

Additional file 1. **Table S1.** The LD between rs9371601 and its nearby SNPs in European and East Asian individuals. (XLSX 249 kb)

Additional file 2. **Table S2.** Association of rs9371601 with nearby gene expression in Brain xQTL dataset. (XLSX 11 kb)

Additional file 3. **Table S3.** Association of rs9371601 with nearby gene expression in GTEx dataset. (XLSX 11 kb)

Additional file 4. **Table S4.** Association of rs9371601 with nearby gene expression in the lymphoblastoid cell lines from 56 European individuals. (DOCX 14 kb)

Additional file 5. **Table S5.** Association of rs9371601 with nearby gene expression in the lymphoblastoid cell lines from 85 East Asian individuals. (DOCX 14 kb)

Additional file 6. **Figure S1.** The transcript map of SYNE1 gene according to data from 1000-Human-Genome website (PDF 368 kb)

Additional file 7. **Table S6.** Association of rs9371601 with the methylation of neabry CpG sites in Brain xQTL dataset. (XLSX 11 kb)

## Data Availability

The datasets used and analyzed during the current study are available from the corresponding author on reasonable request.

## Abbreviations

BPD: Bipolar disorder
CI: Confidence intervals
DLPFC: Dorsal lateral prefrontal cortex
eQTL: Expression quantitative trait loci
GWAS: Genome-wide association study
HWE: Hardy-Weinberg equilibrium
LD: Linkage disequilibrium
meQTL: Methylation quantitative trait loci
OR: Odds ratios
PCR: Polymerase chain reaction
PGC: Psychiatric Genomics Consortium
SNP: Single nucleotide polymorphism

## References

[1] Grande I, Berk M, Birmaher B, Vieta E (2016). Bipolar disorder. Lancet..

[2] Vieta E, Berk M, Schulze TG, Carvalho AF, Suppes T, Calabrese JR (2018). Bipolar disorders. Nat Rev Dis Primers.

[3] Craddock N, Sklar P (2013). Genetics of bipolar disorder. Lancet..

[4] Cichon S, Muhleisen TW, Degenhardt FA, Mattheisen M, Miro X, Strohmaier J (2011). Genome-wide association study identifies genetic variation in neurocan as a susceptibility factor for bipolar disorder. Am J Hum Genet.

[5] Hou L, Bergen SE, Akula N, Song J, Hultman CM, Landen M (2016). Genome-wide association study of 40,000 individuals identifies two novel loci associated with bipolar disorder. Hum Mol Genet.

[6] Ryu E, Nassan M, Jenkins GD, Armasu SM, Andreazza A, McElroy SL (2017). A genome-wide search for bipolar disorder risk loci modified by mitochondrial genome variation. Mol Neuropsychiatry..

[7] Chen DT, Jiang X, Akula N, Shugart YY, Wendland JR, Steele CJ (2013). Genome-wide association study meta-analysis of European and Asian-ancestry samples identifies three novel loci associated with bipolar disorder. Mol Psychiatry.

[8] Ikeda M, Takahashi A, Kamatani Y, Okahisa Y, Kunugi H, Mori N (2018). A genome-wide association study identifies two novel susceptibility loci and trans population polygenicity associated with bipolar disorder. Mol Psychiatry.

[9] Ruderfer DM, Fanous AH, Ripke S, McQuillin A, Amdur RL, Schizophrenia Working Group of the Psychiatric Genomics C (2014). Polygenic dissection of diagnosis and clinical dimensions of bipolar disorder and schizophrenia. Mol Psychiatry.

[10] Muhleisen TW, Leber M, Schulze TG, Strohmaier J, Degenhardt F, Treutlein J (2014). Genome-wide association study reveals two new risk loci for bipolar disorder. Nat Commun.

[11] Ferreira MA, O'Donovan MC, Meng YA, Jones IR, Ruderfer DM, Jones L (2008). Collaborative genome-wide association analysis supports a role for ANK3 and CACNA1C in bipolar disorder. Nat Genet.

[12] FJ MM, Akula N, Schulze TG, Muglia P, Tozzi F, Detera-Wadleigh SD (2010). Meta-analysis of genome-wide association data identifies a risk locus for major mood disorders on 3p21.1. Nat Genet.

[13] Psychiatric Gwas Consortium Bipolar Disorder Working Group (2011). Large-scale genome-wide association analysis of bipolar disorder identifies a new susceptibility locus near ODZ4. Nat Genet.

[14] Xu W, Cohen-Woods S, Chen Q, Noor A, Knight J, Hosang G (2014). Genome-wide association study of bipolar disorder in Canadian and UK populations corroborates disease loci including SYNE1 and CSMD1. BMC Med Genet.

[15] Stahl EA, Breen G, Forstner AJ, McQuillin A, Ripke S, Trubetskoy V, et al. Genome-wide association study identifies 30 loci associated with bipolar disorder. Nat Genet. 2019. 10.1038/s41588-019-0397-8:173062. 10.1038/s41588-019-0397-8 PMC6956732 31043756

[16] Gonzalez S, Gupta J, Villa E, Mallawaarachchi I, Rodriguez M, Ramirez M (2016). Replication of genome-wide association study (GWAS) susceptibility loci in a Latino bipolar disorder cohort. Bipolar Disord.

[17] Zhang X, Zhang C, Wu Z, Wang Z, Peng D, Chen J (2013). Association of genetic variation in CACNA1C with bipolar disorder in Han Chinese. J Affect Disord.

[18] Zeng Z, Wang T, Li T, Li Y, Chen P, Zhao Q (2011). Common SNPs and haplotypes in DGKH are associated with bipolar disorder and schizophrenia in the Chinese Han population. Mol Psychiatry.

[19] Kondo K, Ikeda M, Kajio Y, Saito T, Iwayama Y, Aleksic B (2013). Genetic variants on 3q21 and in the Sp8 transcription factor gene (SP8) as susceptibility loci for psychotic disorders: a genetic association study. PLoS One.

[20] Takata A, Kim SH, Ozaki N, Iwata N, Kunugi H, Inada T (2011). Association of ANK3 with bipolar disorder confirmed in East Asia. Am J Med Genet B Neuropsychiatr Genet.

[21] Wang L, Liu W, Li X, Xiao X, Li L, Liu F (2018). Further evidence of an association between NCAN rs1064395 and bipolar disorder. Mol Neuropsychiatry.

[22] Xie Y, Huang D, Wei L, Luo XJ (2018). Further evidence for the genetic association between CACNA1I and schizophrenia. Hereditas..

[23] Xu W, Liu Y, Chen J, Guo Q, Liu K, Wen Z (2018). Genetic risk between the CACNA1I gene and schizophrenia in Chinese Uygur population. Hereditas..

[24] Yuan J, Hu J, Li Z, Zhang F, Zhou D, Jin C (2017). A replication study of schizophrenia-related rare copy number variations in a Han southern Chinese population. Hereditas..

[25] Zhang L, Zhong X, An Z, Han S, Luo X, Shi Y (2014). Association analysis of the GRM8 gene with schizophrenia in the Uygur Chinese population. Hereditas..

[26] Green EK, Grozeva D, Forty L, Gordon-Smith K, Russell E, Farmer A (2013). Association at SYNE1 in both bipolar disorder and recurrent major depression. Mol Psychiatry.

[27] Rathje M, Waxman H, Benoit M, Tammineni P, Leu C, Loebrich S, et al. Genetic variants in the bipolar disorder risk locus SYNE1 that affect CPG2 expression and protein function. Mol Psychiatry. 2019. 10.1038/s41380-018-0314-z. 10.1038/s41380-018-0314-z PMC6609516 30610203

[28] Loebrich S, Rathje M, Hager E, Ataman B, Harmin DA, Greenberg ME (2016). Genomic mapping and cellular expression of human CPG2 transcripts in the SYNE1 gene. Mol Cell Neurosci.

[29] Cottrell JR, Borok E, Horvath TL, Nedivi E (2004). CPG2: a brain- and synapse-specific protein that regulates the endocytosis of glutamate receptors. Neuron..

[30] Lee MT, Chen CH, Lee CS, Chen CC, Chong MY, Ouyang WC (2011). Genome-wide association study of bipolar I disorder in the Han Chinese population. Mol Psychiatry.

[31] Zhao L, Chang H, Zhou DS, Cai J, Fan W, Tang W (2018). Replicated associations of FADS1, MAD1L1, and a rare variant at 10q26.13 with bipolar disorder in Chinese population. Transl Psychiatry.

[32] Barrett JC, Fry B, Maller J, Daly MJ (2005). Haploview: analysis and visualization of LD and haplotype maps. Bioinformatics..

[33] Purcell S, Neale B, Todd-Brown K, Thomas L, Ferreira MA, Bender D (2007). PLINK: a tool set for whole-genome association and population-based linkage analyses. Am J Hum Genet.

[34] Dupont WD, Plummer WD (1990). Power and sample size calculations. A review and computer program. Control Clin Trials.

[35] Ng B, White CC, Klein HU, Sieberts SK, McCabe C, Patrick E (2017). An xQTL map integrates the genetic architecture of the human brain's transcriptome and epigenome. Nat Neurosci.

[36] GTEx Consortium (2013). The genotype-tissue expression (GTEx) project. Nat Genet.

[37] Stranger BE, Montgomery SB, Dimas AS, Parts L, Stegle O, Ingle CE (2012). Patterns of cis regulatory variation in diverse human populations. PLoS Genet.

[38] Abecasis GR, Auton A, Brooks LD, DePristo MA, Durbin RM, Genomes Project Consortium (2012). An integrated map of genetic variation from 1,092 human genomes. Nature..

[39] Pickrell JK, Coop G, Novembre J, Kudaravalli S, Li JZ, Absher D (2009). Signals of recent positive selection in a worldwide sample of human populations. Genome Res.

[40] Coop G, Pickrell JK, Novembre J, Kudaravalli S, Li J, Absher D (2009). The role of geography in human adaptation. PLoS Genet.

[41] Edwards SL, Beesley J, French JD, Dunning AM (2013). Beyond GWASs: illuminating the dark road from association to function. Am J Hum Genet.

[42] Gamazon ER, Badner JA, Cheng L, Zhang C, Zhang D, Cox NJ (2013). Enrichment of cis-regulatory gene expression SNPs and methylation quantitative trait loci among bipolar disorder susceptibility variants. Mol Psychiatry.

[43] Bray NJ, Hill MJ (2016). Translating genetic risk loci into molecular risk mechanisms for schizophrenia. Schizophr Bull.

[44] Li M, Jaffe AE, Straub RE, Tao R, Shin JH, Wang Y (2016). A human-specific AS3MT isoform and BORCS7 are molecular risk factors in the 10q24.32 schizophrenia-associated locus. Nat Med.

[45] Sekar A, Bialas AR, de Rivera H, Davis A, Hammond TR, Kamitaki N (2016). Schizophrenia risk from complex variation of complement component 4. Nature..

[46] Tao R, Cousijn H, Jaffe AE, Burnet PW, Edwards F, Eastwood SL (2014). Expression of ZNF804A in human brain and alterations in schizophrenia, bipolar disorder, and major depressive disorder: a novel transcript fetally regulated by the psychosis risk variant rs1344706. JAMA Psychiatry.

[47] Huffaker SJ, Chen J, Nicodemus KK, Sambataro F, Yang F, Mattay V (2009). A primate-specific, brain isoform of KCNH2 affects cortical physiology, cognition, neuronal repolarization and risk of schizophrenia. Nat Med.

[48] Hannon E, Spiers H, Viana J, Pidsley R, Burrage J, Murphy TM (2016). Methylation QTLs in the developing brain and their enrichment in schizophrenia risk loci. Nat Neurosci.

[49] Jaffe AE, Gao Y, Deep-Soboslay A, Tao R, Hyde TM, Weinberger DR (2016). Mapping DNA methylation across development, genotype and schizophrenia in the human frontal cortex. Nat Neurosci.

[50] Li M, Luo XJ, Rietschel M, Lewis CM, Mattheisen M, Muller-Myhsok B (2014). Allelic differences between Europeans and Chinese for CREB1 SNPs and their implications in gene expression regulation, hippocampal structure and function, and bipolar disorder susceptibility. Mol Psychiatry.

[51] Chang H, Xiao X, Li M (2017). The schizophrenia risk gene ZNF804A: clinical associations, biological mechanisms and neuronal functions. Mol Psychiatry.

[52] Li M, Chang H, Xiao X (2016). BDNF Val66Met polymorphism and bipolar disorder in European populations: a risk association in case-control, family-based and GWAS studies. Neurosci Biobehav Rev.

[53] Li M, Yue W (2018). VRK2, a candidate gene for psychiatric and neurological disorders. Mol Neuropsychiatry..

[54] Ding Y, Chang LC, Wang X, Guilloux JP, Parrish J, Oh H (2015). Molecular and genetic characterization of depression: overlap with other psychiatric disorders and aging. Mol Neuropsychiatry..

[55] Li L, Chang H, Peng T, Li M, Xiao X (2017). Evidence of AS3MTd2d3-associated variants within 10q24.32-33 in the genetic risk of major affective disorders. Mol Neuropsychiatry..

[56] Pardinas AF, Holmans P, Pocklington AJ, Escott-Price V, Ripke S, Carrera N (2018). Common schizophrenia alleles are enriched in mutation-intolerant genes and in regions under strong background selection. Nat Genet.

[57] Converge consortium (2015). Sparse whole-genome sequencing identifies two loci for major depressive disorder. Nature..

[58] Gandal Michael J., Zhang Pan, Hadjimichael Evi, Walker Rebecca L., Chen Chao, Liu Shuang, Won Hyejung, van Bakel Harm, Varghese Merina, Wang Yongjun, Shieh Annie W., Haney Jillian, Parhami Sepideh, Belmont Judson, Kim Minsoo, Moran Losada Patricia, Khan Zenab, Mleczko Justyna, Xia Yan, Dai Rujia, Wang Daifeng, Yang Yucheng T., Xu Min, Fish Kenneth, Hof Patrick R., Warrell Jonathan, Fitzgerald Dominic, White Kevin, Jaffe Andrew E., Peters Mette A., Gerstein Mark, Liu Chunyu, Iakoucheva Lilia M., Pinto Dalila, Geschwind Daniel H. (2018). Transcriptome-wide isoform-level dysregulation in ASD, schizophrenia, and bipolar disorder. Science.

