# Supplementary material for: Association of SYNE1 locus with bipolar disorder in Chinese population
Source: Hereditas. 2019 Jun 17;156:19. doi: 10.1186/s41065-019-0095-7 (PMC6580462; doi:10.1186/s41065-019-0095-7)
Supplement: Supplementary file 4 — Table S4. Association of rs9371601 with nearby gene expression in the lymphoblastoid cell lines from 56 European individuals. (DOCX 14 kb) [file 41065_2019_95_MOESM4_ESM.docx]

**Table S4. Association of rs9371601 with nearby gene expression in the lymphoblastoid cell lines from 56 European individuals**

|  | **Beta estimate** | **Standard error** | **T-value** | **P-value** |
| --- | --- | --- | --- | --- |
| *ESR1* | 0.000667 | 0.0206 | 0.0325 | 0.974 |
| *SYNE1* | 0.0131 | 0.0199 | 0.660 | 0.513 |
| *MYCT1* | -0.0123 | 0.0298 | -0.412 | 0.683 |
| *VIP* | -0.00572 | 0.0142 | -0.404 | 0.688 |
