# Supplementary material for: Association of SYNE1 locus with bipolar disorder in Chinese population
Source: Hereditas. 2019 Jun 17;156:19. doi: 10.1186/s41065-019-0095-7 (PMC6580462; doi:10.1186/s41065-019-0095-7)
Supplement: Supplementary file 5 — Table S5. Association of rs9371601 with nearby gene expression in the lymphoblastoid cell lines from 85 East Asian individuals. (DOCX 14 kb) [file 41065_2019_95_MOESM5_ESM.docx]

**Table S5. Association of rs9371601 with nearby gene expression in the lymphoblastoid cell lines from 85 East Asian individuals**

|  | **Beta estimate** | **Standard error** | **T-value** | **P-value** |
| --- | --- | --- | --- | --- |
| *ESR1* | -0.00529 | 0.0175 | -0.302 | 0.764 |
| *SYNE1* | 0.00266 | 0.0101 | 0.263 | 0.793 |
| *MYCT1* | -0.00453 | 0.0160 | -0.283 | 0.778 |
| *VIP* | 0.0150 | 0.0125 | 1.196 | 0.236 |
