# Supplementary figures and images for: Association of SYNE1 locus with bipolar disorder in Chinese population
Source: Hereditas. 2019 Jun 17;156:19. doi: 10.1186/s41065-019-0095-7 (PMC6580462; doi:10.1186/s41065-019-0095-7)

Figure S1. The transcript map of SYNE1 gene according to data from 1000-Human-Genome website

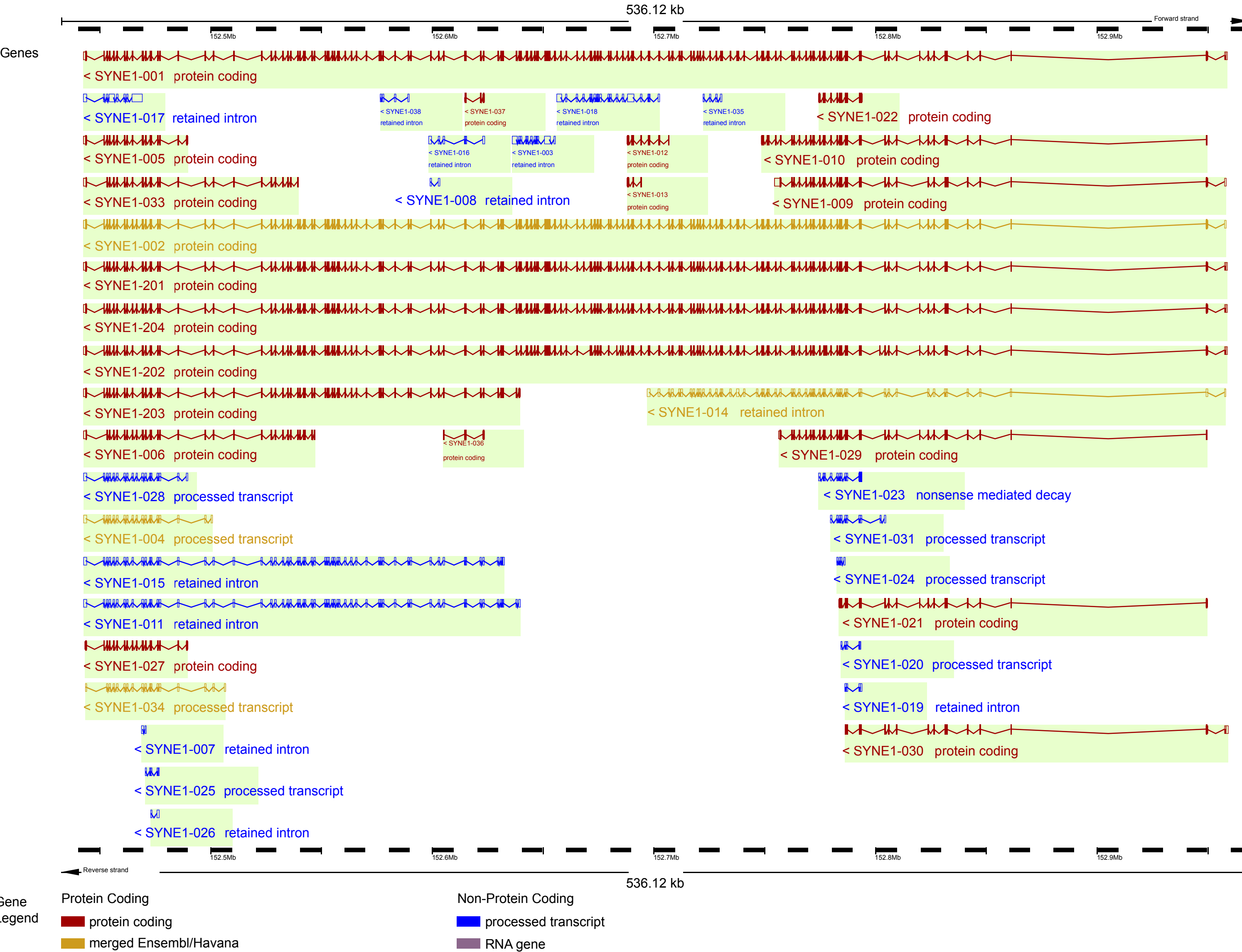

Supplement: Supplementary file 6 — Figure S1. The transcript map of SYNE1 gene according to data from 1000-Human-Genome website (PDF 368 kb) [file 41065_2019_95_MOESM6_ESM.pdf]
